# The Role of Ion Channels in Functional Gastrointestinal Disorders (FGID): Evidence of Channelopathies and Potential Avenues for Future Research and Therapeutic Targets

**DOI:** 10.3390/ijms241311074

**Published:** 2023-07-04

**Authors:** Fatima Maqoud, Domenico Tricarico, Rosanna Mallamaci, Antonella Orlando, Francesco Russo

**Affiliations:** 1 Functional Gastrointestinal Disorders Research Group, National Institute of Gastroenterology IRCCS “Saverio de Bellis”, Castellana Grotte, 70013 Bari, Italy; fatima.maqoud@irccsdebellis.it (F.M.); antonella.orlando@irccsdebellis.it (A.O.); 2 Section of Pharmacology, Department of Pharmacy-Pharmaceutical Sciences, University of Bari Aldo Moro, 70125 Bari, Italy; domenico.tricarico@uniba.it; 3 Department of Biosciences, Biotechnologies and Environment University of Bari Aldo Moro, 70125 Bari, Italy; rosanna.mallamaci@uniba.it

**Keywords:** ion channels, functional gastrointestinal disorders, transporters, transient receptor potential, aquaporin, ATP-sensitive K^+^ channels, calcium-activated K^+^ channels, voltage-dependent sodium channels, irritable bowel syndrome (IBS), intestinal dysmicrobism

## Abstract

Several gastrointestinal (GI) tract abnormalities, including visceral hypersensitivity, motility, and intestinal permeability alterations, have been implicated in functional GI disorders (FGIDs). Ion channels play a crucial role in all the functions mentioned above. Hormones and natural molecules modulate these channels and represent targets of drugs and bacterial toxins. Mutations and abnormal functional expression of ion channel subunits can lead to diseases called channelopathies. These channelopathies in gastroenterology are gaining a strong interest, and the evidence of co-relationships is increasing. In this review, we describe the correlation status between channelopathies and FGIDs. Different findings are available. Among others, mutations in the *ABCC7*/CFTR gene have been described as a cause of constipation and diarrhea. Mutations of the *SCN5A* gene are instead associated with irritable bowel syndrome. In contrast, mutations of the *TRPV1* and *TRPA* genes of the transient receptor potential (TRP) superfamily manifest hypersensitivity and visceral pain in sensory nerves. Recently, mice and humans affected by Cantu syndrome (CS), which is associated with the mutations of the *KCNJ8* and *ABCC9* genes encoding for the Kir6.1 and SUR2 subunits, showed dysfunction of contractility throughout the intestine and death in the mice after the weaning on solid food. The discovery of a correlation between channelopathies and FIGD opens new avenues for discovering new direct drug targets for specific channelopathies, leading to significant implications for diagnosing and treating functional GI diseases.

## 1. Introduction

Channelopathies refer to diseases caused by the dysfunction/mutation of ion channel gene-encoding subunits or their interacting proteins.

Encoded by more than 400 genes, ion channels and aquaporin water channels (AQPs), mainly localized in the lipid bilayer, play a crucial role in the transit of ions and water through a semipermeable lipid bilayer. Ion channels are expressed in all cells and are critical to the specialized functions of many cells [1]. Aquaporins (AQPs) are widely localized on specific cell types in various organs and tissues; they play a crucial role in the management of transcellular fluid movement [2]. In the gastrointestinal (GI) epithelium, ion channels and AQPs are expressed throughout the gut. They are involved in a large part of intestinal functions, and their dysfunction contributes to the symptomatology of functional bowel disease. Ion channels in epithelial cells and intestinal smooth muscle cells drive most aspects involved in digestion, including fluid secretion, absorption, motility, and intestinal permeability. Furthermore, sensory signals, including aberrant pain and visceral sensation, depend on the expression and dysfunction of receptors and ion channels at the level of extrinsic sensory neurons [3].

Alterations in the ion channels are caused by defects of genes encoding for the ion channel subunits, including some autoimmune diseases that cause a group of diseases named “channelopathies”. These diseases are often caused by faults in channel function or expression related to a wide range of genomic, transcriptional, translational, or posttranslational modifications such as phosphorylation, ubiquitination, glycosylation, and palmitoylation, regulated by noncoding RNAs or interactions of multiprotein complexes with subunits that form the ion channel. Furthermore, dysregulations in ion channel modulation by multiple signaling molecules, such as cyclic nucleotides and lipids, can lead to channelopathies [4]. Channelopathies are well established in the fields of cardiology and neurology [5,6,7], but their role is gaining much interest also in gastroenterology due to their multiple actions in the control of GI functions [8,9].

In GI diseases, channelopathies can contribute to the development of various functional GI disorders (FGIDs) [8]. The term “FGIDs” encompasses a group of disorders that affect the function of the digestive system but do not have a clear structural or biochemical basis. These disorders can be categorized into irritable bowel syndrome (IBS), functional dyspepsia (FD), and functional constipation [10].

**Figure 1:**
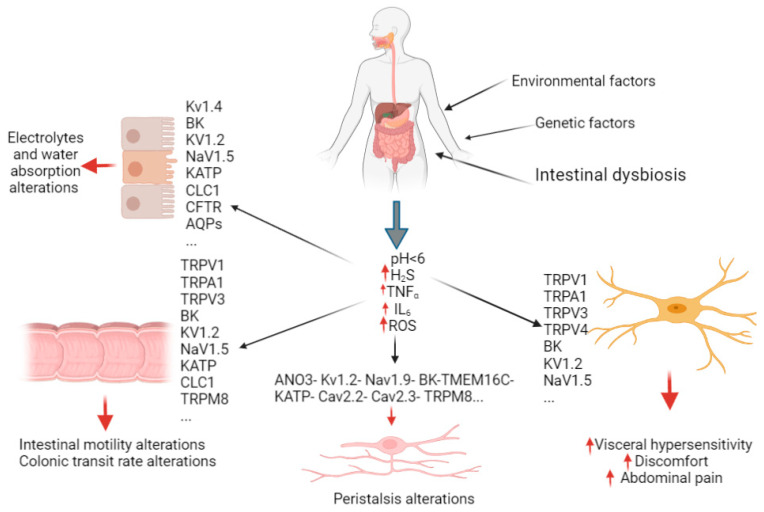
Illustrative diagram of the possible involvements of ion channels and AQPs in FGIDs. Various distinct ion channels or subtypes can target diverse GI cells to regulate some crucial GI activities, including fluid secretion, absorption, motility, intestinal permeability, and sensory signals management, and their dysfunction contributes to the symptomatology and the pathophysiological process of FGID. Some ion channels are associated with hypersensitivity and abdominal pain, such as the transient receptor potential (TRP) families. The metabolic sensing ion channels, such as the ATP-sensitive (KATP) and the calcium channels (Cav), regulate the peristaltic movements. The chloride channels (CLC), TRP, and sodium channels (Nav) are involved in the regulation of intestinal motility, while the cystic fibrosis transmembrane regulators (CFTRs), chloride channels, several potassium channels (BK, Kv, and KATP), and aquaporins (AQPs) regulate electrolyte absorption and water. These regulations are mediated by inflammatory factors and pH.

Multiple pathophysiological mechanisms are involved in the manifestation of FGIDs (Figure 1) [10]. Chronic infections, altered intestinal microbiota, low-grade mucosal inflammation, and fermentation and osmotic effects are all mechanisms evoked in the FGIDs etiology [11]. Moreover, alterations in gut dysmotility, intestinal barrier dysfunction, gut immune dysfunction, visceral hypersensitivity, altered GI secretion, and the presence and degree of bile acid are some pathophysiological mechanisms involved in FGIDs [12]. In these processes, ion channels play essential roles, and the disruptions of their ability to conduct ions can lead to FGID pathophysiology [8].

In this review, through a stratified literature search in the electronic databases PubMed, Virtual Library in Health (BVS), and NCBI using the search terms “ion channels” or “receptor” or “aquaporin water channels (AQP)” and “visceral hypersensitivity” or “visceral nociception” or “tight junctions interactions” or “colon motility” or “colonic fluid and electrolyte transport” and “syndrome of irritable bowel IBS/FGID”, we tried to investigate the involvement of ion channels and AQP in different aspects related to IBS.

We also evaluated the possibility of the study of genes coding for ion channels and AQP as diagnostic, prognostic, or therapeutic targets for low-grade GI disorders/IBS and inflammation (Table 1). These disorders involve excitable and non-excitable cells such as neurons and smooth muscle cells.

Electrically excitable and non-excitable cells are distinct cell types found in living organisms, differing in their capacity to generate and propagate electrical signals, specifically action potentials. Electrically excitable cells, primarily located in the nervous system (neurons) and muscle tissues (cardiac muscle cells and skeletal muscle fibers), possess unique abilities. Notable characteristics of electrically excitable cells include maintaining the resting membrane potential and an electrical charge difference across the cell membrane, where the interior is typically negatively charged compared to the exterior. Electrically excitable cells also have specialized ion channels in their membranes, regulating the flow of ions such as sodium (Na^+^), potassium (K^+^), and calcium (Ca^2+^), which play a critical role in generating and transmitting action potentials. Conversely, non-excitable cells cannot generate and transmit action potentials or electrical signals. These cells are present in various tissues and organs, including epithelial cells, connective tissue cells, and most circulatory and digestive systems cells. Key features of non-excitable cells involve the maintenance of a stable resting membrane potential without rapid fluctuations observed in excitable cells. Instead of action potentials, non-excitable cells regulate their physiological processes through alternate signaling mechanisms such as chemical messengers such as hormones, paracrine signals, or mechanical stimuli [13].

While non-excitable cells may possess ion channels, their role differs from excitable cells. Ion channels in non-excitable cells predominantly regulate cellular functions such as fluid balance, nutrient transport, and cell-to-cell communication rather than generating electrical signals. It is worth noting that specific cells exhibit a hybrid behavior, combining features of both excitable and non-excitable cells. For instance, cardiomyocytes in the heart display electrical excitability to neurons and muscle cells, albeit differing in specific aspects of their ion channels and signaling mechanisms. Ca^2+^ ions are essential in regulating the electrical activity and contractility of the tissues [14]. In cells with voltage-gated calcium channels, the increase in intracellular calcium ions occurs due to cell depolarization, which activates these calcium channels. On the other hand, the reduced activity of TRP channels leads to cell hyperpolarization, causing an increase in the electrochemical gradient, which, in turn, leads to an elevation of intracellular calcium ions. However, if the cell expresses both functional voltage-gated calcium channels and TRP channels, the activation of TRP channels will depolarize the cell, thereby activating the voltage-gated calcium channels and resulting in an increase in intracellular calcium ions [15].

**Table 1:** The role of ion channels in FGIDs, literature data.

Channel Class	Gene	Channel Protein	Type of Defect	Impact in IBS	Ref.
Chloride channels	*CLCA1*	CLCA1	SNP	IBS risk factor increases; roles in intestinal fluid secretion and secretory diarrhea	[16]
	*CLCA2*	CLCA2			
	*CLCA4*	CLCA4			
	*ANO3*	TMEM16C		Control of gut peristalsis mediated by the interstitial cells of Cajal	[16,17]
	*CLCN2*	CLC-2	Genetic variants/differential gene expression (downregulation)	Promotes GI inflammation and tumorigenicity	[16]
	*SLC26A3*	SLC26A6, PAT1			
	*CLCN3*	CLC-3			
	*ABCC7*	CFTR	SNP/genetic variants		[15,16]
Potassium channels	*KCNA4*	Kv1.4	SNP	Electrolyte secretion and absorption dysfunction	[14,16]
	*KCNJ4*	Kir2.3	SNP/genetic variants	GI motility and dysmotility syndromes	
	*KCNJ8/* *ABCC9*	KATP	Genetic variants (gain-of-function mutant (GoF))	GI motility and dysmotility syndromes in patients CS	[18,19]
	*KCNJ8*	Kir6.1	Genetic variants/differential gene expression (downregulation)	Electrolytes secretion and absorption dysfunction	[20]
	*KCNA2*	Kv1.2	Differential gene expression (upregulation)	Increases the excitability of colonic DRG neurons and consequently increases visceral hypersensitivity	[21,22]
	*KCNMA1*	hSlo/BK	Differential gene expression (upregulation)	Increased visceral hypersensitivity	[23]
Sodium channels	*SCN5A*	Nav1.5	Genetic variants (Loss-of function)	SCN5A missense mutations in 2.2% of patients with diarrhea-predominant IBS	[24,25,26,27]
	*SCN2A*	Nav1.2	Genetic variants	Severe GI symptoms	[28]
	*SCN11A*	Nav 1.9	Genetic variants (gain-of-function mutation)	Increased electrical activity with altered membrane potential in myenteric neurons resulting in increased discomfort, abdominal pain, and diarrhea	[29]
Calcium channels	*CACNA1E*	Cav2.3 type R		GI sensorimotor development and function, visceral sensation and GI motility Spasmolytic effects and inhibition of GI contractility are associated with slower colonic transit rates and increased risk of IBS with constipation	[17,30]
	*CACNA1A*	Cav2.1, EA2/N-type	SNP/genetic variants/polymorphisms	GI sensorimotor development and function, visceral sensation and GI motility	[14,27]
	*CACNA1S CACNA1C CACNA1D CACNA1F*	Cav1 channels (1.1–1.4)/L-type	Differential gene expression (upregulation)	Colonic motility dysfunction	[31]
TRP channels	*TRPV3*	TRPV3	SNP/genetic variants, Differential gene expression (upregulation)	Intestinal chemosensitivity and abdominal pain	[32]
	*TRPM8*	TRPM8	SNP/genetic variants/polymorphisms	TRPM8 polymorphisms are associated with slower colonic transit and increased risk of IBS-C and IBS-M TRPM8 agonists (L-menthol) decrease IBS pain symptoms and reduce the release of inflammatory cytokines IL-1β, IL-6, and TNF-α	[27,30]
	*TRPV1*	TRPV1	Differential gene expression (upregulation)/genetic polymorphism	Its activation is probably with acid pH (pH < 6) and other endogenous agonists (reactive oxygen species (ROS), adenosine, ATP, polyamines (e.g., spermine, spermidine, and putrescine) characteristic of the colon in patients with IBS and increased visceral sensitivity, GI dysfunction, and functional dyspepsia (FD)) (Figure 1)	[29,31,32,33]
	*TRPA1*	TRPA1	Differential gene expression (upregulation) (Figure 1)	Activated by hydrogen sulfide, levels of which are higher in IBS-D patients due to gut dysbiosis, it is likely to act as a directly mechanosensitive nociceptor in hyperalgesia Modulated by several endogenous agonists (e.g., prostaglandins, reactive oxygen species (ROS), cytokines (e.g., TNF-α and IL-6), bradykinin and hydrogen sulfide (Figure 1).	[33,34,35,36,37,38]
	*TRPV4*	TRPV4	Elevated levels of endogenous 5,6-epoxyeicosatrienoic acid (5,6-EET) agonist in colonic biopsy supernatant from patients with IBS-D	Increased intestinal mechanosensitive nociceptor and visceral hypersensitivity	[39,40]
	*AQP4*	AQP4	Differential gene expression (downregulation)	Water secretion and absorption dysfunction.	[41]
AQP channels	*AQP3*	AQP3	Differential gene expression (downregulation) in patients with IBS-D	Deferential expression of AQP in IBS patients is related to colonic absorptive dysfunction and can cause impaired water absorption, loose stools, and diarrhea or constipation	
	*AQP7*	AQP7	Differential gene expression (upregulation) in patients with IBS-D	Deferential expression of AQP in IBS patients is related to colonic absorptive dysfunction and can cause impaired water absorption, loose stools, and diarrhea or constipation	
	*AQP8*	AQP8	Differential gene expression (upregulation) in patients with IBS-D		

## 2. Ion Channels and FGIDs

### 2.1. Ion Channels and Irritable Bowel Syndrome

IBS is a GI disorder causing pain, discomfort, bloating, and bowel habit changes. Stress and anxiety can alter gut motility, visceral perception, and immune function, contributing to IBS development [29,30]. Dysbiosis or gut microbiota composition changes may also contribute to IBS symptoms and pathogenesis [32]. Studies have identified several genes associated with an increased risk of IBS, including those regulating gut motility, inflammation, and pain perception [32].

Although IBS’s exact cause is unknown, it is thought to involve a complex interplay of several mechanisms linking the altered gut microbiome/dysbiosis to altered brain–gut interactions [33]. Bowel motility and secretory dysfunctions, enhanced visceral sensitivity (VH), altered pain perception, increased gut mucosal immune activation, and somatic and psychiatric comorbidities are also involved [42].

VH is characterized by abnormal responses to normal physiological stimuli (known as allodynia) and a heightened perception of pain in the abdominal area (known as hyperalgesia). VH is believed to be the main contributor to the pain experienced by individuals with irritable bowel syndrome (IBS) [43]. This heightened sensitivity is not solely attributed to changes in motor activity but is likely influenced by various factors related to inflammation and stress, leading to the sensitization of both peripheral and central nociceptive pathways [44,45]. Recently, there has been increasing interest in exploring the connection between channelopathies and IBS.

Mutations in the *KCNQ1* gene encoding the potassium channel Kv7.1 have been associated with an increased risk of developing IBS. Furthermore, IBS patients were more likely to have mutations in *KCNQ1* than healthy individuals [46]. In a case report of a pediatric patient with a deleterious genetic mutation in the KCNQ1 Ser 349 Ter gene consistent with LQT1, GI symptoms were reported, including widespread abdominal pain, vomiting, and diarrhea in the absence of common gastrointestinal pathogens, which were all negatives [47].

Furthermore, in some individuals with Jervell and Lange-Nielsen syndrome (JLNS; with nonsense mutations KCNQ1 p.Arg518X and p.Arg190AlafsX95) with long QT type 1, serum gastrin levels are significantly elevated, accompanied by absent acid secretion and presence of multiple gastric carcinoid tumors. This evidence suggests the role of the KCNQ1 gene in gastric secretion and gastrin as a marker of arrhythmia severity [48,49].

Conversely, many patients with cardiac arrhythmias caused by mutations in SCN5A, encoding the α-subunit of the voltage-gated sodium channel NaV1.5, also have symptoms of irritable bowel syndrome (IBS) [26]. In addition, epileptic patients carrying mutations in SCN1A, SCN2A, SCN8A, KCNB1, KCNQ2, and KCNQ3 (encoding the α-subunit of the voltage-gated sodium channels Nav1.1, Nav1.2, Nav1.6, Kv 2.1, Kv 7.2, and Kv 7.3 respectively) have severe GI symptoms, and some patients require supplemental feeding via nasogastric or gastrostomy tube [50,51]. These data raise the question that the proteins encoded by these genes may contribute are specifically involved in the proper function of the GI tract, including controlling contractile activity in the intestinal tract and mediating colonic afferent sensory responses to noxious stimuli.

In addition to these genetic studies, research has been addressed to investigate the pharmacological effects of channel blockers on IBS symptoms. Among them, nifedipine, a L-type calcium channel blocker, improved symptoms in patients with IBS. In patients with IBS, administering nifedipine (20 mg sublingually) reduced the postprandial increase in both myoelectric and colonic contractile activity and motility index. The study suggested that the L-type calcium channel blocker may have reduced pain and improved motility in the GI tract [52,53].

In a clinical study of 91 patients with diarrhea-predominant IBS, treatment with either pinaverium bromide L-type calcium channel blocker with specificity to GI tract smooth muscle (50 mg, t.i.d.) or mebeverine (100 mg, t.i.d.) for 2 weeks resulted in decreased stool frequency with improvement in global sensation in patients with diarrhea-predominant IBS. Treatment with both pinaverium bromide and mebeverine was unable to relieve abdominal pain, suggesting that calcium channels are closely related to the functioning of epithelial cells and smooth muscle cells [54].

### 2.2. Ion Channels and Functional Dyspepsia

Functional dyspepsia (FD) is a common digestive disorder that causes persistent or recurrent upper abdominal pain, discomfort, and other symptoms such as early satiety, postprandial fullness, and bloating without underlying structural abnormalities. Its etiological mechanisms involve physiological, psychological, environmental, and genetic factors [55]. Gastric motility and emptying, visceral hypersensitivity, and gastric acid secretion abnormalities have been associated with FD development [56]. Stress, anxiety, and dietary habits are among the psychological and environmental factors [57]. Genetic factors have also been identified. Several genes have been identified that are associated with an increased FD risk, including genes involved in the regulation of gastric motility and secretion, inflammation, and pain perception [58]. Gut microbiota alterations, including decreased beneficial bacteria and increased content of pathogenic bacteria (i.e., *Bifidobacterium* or *Helicobacter pylori*) [59], have also been suggested as contributors.

The relationship between channelopathies and FD has been investigated in different studies. Mutations in the *SCN5A* gene, which encodes the sodium channel Nav1.5, were associated with an increased risk of developing FD. The study found that individuals with FD were more likely to have mutations in *SCN5A* than individuals without FD [8,60].

In addition to these genetic studies, the effects of channel blockers on FD symptoms have also been considered in the past years [8,61].

Since calcium-channel blocking agents can regulate GI tract function by reducing smooth muscle contraction and inhibiting cellular secretion, previous studies have mainly focused on the ability of these agents to impede smooth muscle contraction [62]. Verapamil and diltiazem have been shown to reduce contractions in esophageal smooth muscle in animals such as opossums and baboons, resulting in reduced peristalsis amplitude and lower esophageal sphincter pressure. Similarly, human studies have demonstrated that oral doses of diltiazem and nifedipine have similar effects on the esophagus, with nifedipine now largely being used for treating achalasia [63]. However, no experimental evidence supports these drugs’ significant effects on FD treatments.

### 2.3. Ion Channels and Functional Constipation

Functional constipation (FC) is a type of chronic constipation not caused by any underlying medical condition or structural abnormality in the digestive tract. It is a common condition affecting children and adults worldwide, and it is estimated that about 14% of the general population suffers from functional constipation at some point in their lives [64].

The exact cause of functional constipation is not well understood. However, several factors have been proposed to contribute to its development, including lifestyle and dietary habits, gut microbiota, and psychosocial factors [65].

Lifestyle factors, such as physical inactivity and sedentary behavior, increase the risk of developing FC [66]. Similarly, dietary factors, such as low fiber intake and high intake of processed and refined foods, have been associated with an increased risk of constipation [67].

The gut microbiota also plays an essential role in developing FC. Studies have shown that alterations in the gut microbiota composition and function can contribute to the development of constipation [68]. For example, a decrease in *Bifidobacterium* and *Lactobacillus* species content and an increased abundance of pathogenic bacteria, such as *Clostridium difficile*, have been observed in FC patients [69].

Psychosocial factors, such as stress, anxiety, and depression, can also contribute to FC development. Stress and anxiety can alter the gut–brain axis, leading to gut motility and secretion changes, which can contribute to IBS and constipation [70].

The scientific literature has long debated whether FC arises from enteric neuropathy and whether genetic factors play a role in its development. This discussion has been ongoing since the 1960s, primarily based on clinical observations of early-onset symptoms and familial patterns. However, upon closer examination of this literature, little to no proof of Mendelian inheritance, unafflicted monozygotic twins, and comparable rates of family history in community controls can be found [71]. There has recently been growing interest in the relationship between channelopathies and FC.

One ion channel implicated in functional constipation is the sodium channel Nav1.5, found in the colon’s smooth muscle cells. Studies have shown that alterations in Nav1.5 expression or function may contribute to abnormal colonic motility and constipation. Mutations in the *SCN5A* gene, which encodes the sodium channel Nav1.5, have been identified as potential genetic risk factors for IBS and functional constipation [72].

Another ion channel associated with functional constipation is the calcium-activated chloride channel (CaCC). The chloride channel family has a role in fluid viscosity regulation and is modulated by second messengers [73]. Studies have shown that CaCCs play a crucial role in fluid secretion into the intestinal lumen, which is necessary for normal bowel movements. Dysfunctional CaCCs may impair fluid secretion, leading to constipation [74].

From a therapeutic point of view, the effects of channel modulators have also been evaluated on FC symptoms. A recent study [61] reported that a potassium channel opener called pinacidil improved symptoms in patients with FC. The study suggested that the potassium channel opener may have improved colonic motility and reduced abdominal pain in patients with FC. Furthermore, most recent drugs used for FC and constipation-predominant inflammatory bowel syndrome (IBS-C) primarily target ion transport pathways to increase epithelial secretion [8]. Among these drugs, lubiprostone is an agonist of the chloride channel encoded by the *CLC2* gene [75], and linaclotide also acts by stimulating the cystic fibrosis transmembrane conductance regulator (CFTR) via an increase in cGMP secondary to activation of guanylate 2C encoded in the *GUCY2C* gene [76].

## 3. Ion Channels and the Pathophysiology of FIGDs

### 3.1. Ion Channels and Tight Junctions Alterations

Tight junctions (TJs) are unique organelles in epithelial cells located in the apical–lateral region and play crucial roles in the transport functions of epithelial cells. The two primary tight junction functions are the gate and the fence function.

The existence of alterations in the expression and distribution of TJ proteins in IBS patients has been widely documented. Decreased occludin, claudin-1, and claudin-4 expression were found in colonic biopsies of IBS patients, especially those with diarrhea-predominant subtype [77]. Increased expression of claudin-2 has been detected in the colonic mucosa of IBS patients [78], which may lead to increased paracellular permeability.

TJs regulators such as cytokines, growth factors, and neurotransmitters have also been implicated in IBS pathogenesis [79]. In a recent study by Awad et al. [80], they observed a decrease in occludin expression, which caused tricellulin to become delocalized from the tricellular TJ. This delocalization, in turn, increased macromolecular permeability, contributing to the influx of antigens into the mucosa. As a result, a low-grade inflammatory process persisted in patients with IBS.

Moreover, Horie et al. [81] showed that 5HT reduces occludin expression without affecting the expression of other TJ-associated proteins. The mechanism behind this phenomenon is unclear, but the authors hypothesized that the serotonin-induced oxidative stress might modify the amino-acid residues of occludin, reducing occludin expression.

The presence of multiprotein complexes consisting of transmembrane and peripheral membrane proteins at the level of the TJ makes the latter dynamic structures. The process of paracellular transport that occurs via TJ is tightly regulated in a complex manner via various extracellular and intracellular signals. The TJ permeability is controlled by multiple factors, including ions and their transporters [26].

Physiological and pathological factors, comprising growth factors (EGF, HGF, VEGF, FGF, and TGF-β), cytokines, hormones, drugs, and nutrients [5,26,46,82], can alter the barrier properties of tight junctions acutely and in the long term. In several diseases (e.g., chronic inflammatory diseases such as inflammatory bowel disease, multiple sclerosis, allergies, microbial infectious diseases, diabetes and its complications, and cancer) [46,77,83], dysregulation of the barrier function of epithelial and endothelial TJs may occur. There are many factors underlying the regulation or dysregulation of TJ functionality. However, several studies have shown that different transporters and ion channels have a function to modulate the structure and paracellular permeability at the level of tight junctions, which is regulated by ion channels and pumps such as the Na, K-ATPase that catalyzes the transport of three sodium ions and two potassium ions into the cell per pump cycle in an ATP-dependent manner, thus generating sodium and potassium gradients across the plasma membrane, which is required for the epithelial polarization. This event plays a crucial role in TJ formation [84]. The Na, K-ATPase collaborates synergistically with the cell adhesion molecule E-cadherin in assembling TJs [66,67,68,69,70,71,72,73,74,75,76,77,78,79,80,81,82,83,84,85,86].

Recent studies have also suggested that ATP-sensitive K^+^ (KATP) channels regulate epithelial TJ permeability [21,87]. KATP channels are essential in coupling the cellular metabolic state to electrical activity [5,87,88,89]. KATP channels are hetero-octameric protein complexes composed of two Kir6.1 subunits encoded by the *KCNJ8* and *KCNJ11* genes, respectively, and the sulfonylurea receptor (SUR1 and 2) subunits, encoded by the *ABCC8* and *ABCC9* genes, respectively, belonging to the ABC transporter superfamily such as the CFTR. Kir6.1/SUR2A complexes are restricted to TJs in human gastric mucosa; they co-immuno-precipitate and colocalize with occludin in the liver [88].

Mice and humans affected by Cantu syndrome (CS), which is associated with the mutations of the *KCNJ8* and *ABCC9* genes encoding the Kir6.1 and SUR2 subunits, showed dysfunction of contractility throughout the intestine and death in the mice after the weaning on solid food [21]. The KATP channels are modulated by hormones such as insulin and second messengers and are targets of drugs of therapeutic interest, such as antidiabetic and cardiovascular drugs, amino acids, or bacterial toxins such as staurosporine [90,91] that may affect the contractility of the intestine. In vivo studies in rats demonstrated that the KATP channel antagonist tolbutamide caused an increase in tight junction permeability. In contrast, the agonist diazoxide caused a decrease in paracellular permeability in the small intestine [21]. Staurosporine has recently been associated with sarcopenia in Asian people with intestinal dysmicrobism based on the abundance of *Desulfovibrio piger* producing this toxin [92].

Other studies demonstrated that CLC-2 agonism, using lubiprostone, induced rapid recovery of transepithelial electrical resistance and significantly reduced paracellular permeability in the ischemia-injured porcine intestinal mucosa [93].

Some studies have shown that TRPV4, a transient receptor potential (TRP) channel superfamily member, is co-distributed with E-cadherin at the epithelial cell lateral plasma membrane level. Furthermore, its activation caused a break of the tight junction filaments and decreased expression claudin-4 [30].

### 3.2. Ion Channels in the Visceral Hypersensitivity and Motility Alterations

The stratified research shows strong links between ion channel dysfunction and AQPs with visceral hypersensitivity in IBS (Table 1). The voltage-gated sodium (Nav) [46,83], calcium (Cav), specifically Cav3.2 [8], transient receptor potential vanilloid TRPV1, TRPV3, TRPV4, and TRPA1 [45,77], voltage-gated sodium (Nav) (Nav1.1, Nav1.3, Nav1.5, Nav1.6, Nav1.7, Nav1.8, and Nav1.9) [5,94], and big/large-conductance Ca^2+^-activated K (BKCa) channels [23,95] are involved in the genesis of visceral hypersensitivity in IBS.

BK channels are known for their roles in regulating hyperkalemic states [96], epilepsy, alcohol dependence, and cell proliferation [97,98] in brain tumors, as well as in the vascular tone regulation by estrogen [99]. BK channel subunits are functionally coupled to carbonic anhydrase enzymes in renal epithelial cells, neurons, and skeletal muscle. The enzymes are expressed in the intestine and bacterial microorganisms and have a role in intestinal dysmicrobism-associated diseases [100]. For instance, bacterial carbonic anhydrase provides the indispensable CO_2_ and HCO_3_^−^/protons to microbial biosynthetic pathways. Thus, their inhibition might impair the survival of microbes, but the human genome encodes only for *α*-enzyme, which is phylogenetically and structurally well separated by the bacterial *β*- and *γ*-enzymes. The BK channel alpha subunit shares a common molecular area of interaction with human carbonic anhydrase enzymes and is a drug target of carbonic anhydrase inhibitors, natural glycosidic steroid-based molecules [101,102], and flavonoids regulating cell proliferation [62]. The reduced expression activity of carbonic anhydrase-1 proteins is associated with fatigue [103,104]. Furthermore, these proteins were recently found to be particularly elevated in the serum of IBS patients, thus representing a promising biomarker.

It should be noted that the Nav1.5 channel has a crucial role in adequately functioning intestinal smooth muscle cells and interstitial cells of Cajal [27]. Mutations in *SCN5A*, which encodes the pore-forming α subunit of Nav1.5, have been found in patients with IBS and functional dyspepsia [8]. It is estimated that 2% of patients with IBS carry mutations in *SCN5A*, most of which are loss-of-function mutations and are primarily associated with constipation, in contrast to diarrhea-predominant subtypes in patients with IBS [26,27].

Thus, channelopathies represent potential abnormalities underlying GI dysfunction and IBS. In an evaluation study of the expression profile and variants of the genes coding for ion channels in subjects with IBS compared to healthy subjects, the pathophysiological involvement of several genes in the processes related to IBS was highlighted, including Nav1.8, Nav1.1, Nav1.2, Nav1.7, BK, Kir6.1, AQP3, AQP4, AQP7, AQP8, TRPM2, TRPM3, TRPM8, TRPV1, TRPV3, and TRPV4 [30,32].

In addition, ion channels are directly involved in mechanotransduction (TRPV1, TRPA1, TRPV3, TRP4, Kv7.1, and BK); hence, encoding colorectal mechanical stimuli by sensory afferents is crucial for the mechanisms underlying related visceral pain in IBS patients [84]. The TRPV1 channel is a sensor of nociception in different tissues. It is regulated by hormones such as oxytocin that directly bind to the TRPV1 sites [105,106], is modulated by several natural molecules mediating the nociception, and is a target of novel analgesic drugs under development. It also regulates cell proliferation [107,108].

### 3.3. Ion Channels and Intestinal Permeability

Increased intestinal permeability (IP) refers to the increased passage of molecules and substances into the bloodstream through the intestinal epithelial barrier. This function is essential for maintaining a proper balance between nutrient absorption and excluding harmful substances from the gut [109].

IBS patients often show an increased IP, which can lead to the translocation of luminal bacteria and their products into the systemic circulation, triggering immune and inflammatory responses [110]. The relationship between increased intestinal permeability and IBS is complex and likely bidirectional. While increased permeability may contribute to IBS pathogenesis, IBS symptoms and their associated stress and inflammation can also affect IP. Moreover, the relationship between increased permeability and IBS subtypes (such as diarrhea-predominant, constipation-predominant, or mixed) may differ, suggesting distinct underlying mechanisms [111,112].

Increased IP has been linked to generating symptoms in IBS [113], including VH [85]. Ion channels are directly involved in regulating intestinal motility and spontaneous contractions. Several research groups related the dysfunction of ion channels and AQP channels to the manifestation of permeability and motility dysregulation in the IBS pathophysiology.

Amato et al. [86] demonstrated that TRPM8 receptors are expressed in the human distal colon under healthy conditions, and that ligand-dependent activation of TRPM8 can reduce spontaneous colonic motility, probably through the opening of large-conductance Ca^2+^-activated K^+^ channels, thereby contributing to the onset of pain in IBS patients [34,114].

Furthermore, AQPs are expressed in most tissues and are associated with several physiological and pathophysiological processes.

The movement through AQPs occurs through a shared passive process, yet the control and arrangement of AQPs differ depending on the specific cell type and tissue. AQPs play a role in regulating cell volume, the flow of water between cells, and water balance, affecting the surface expression of the membrane proteins, promoting cell adhesion, and enabling swift water transportation across various cell membranes mediated by specialized channels [115,116].

In the literature, several AQP channels have been detected in human colonic epithelial cells (AQP1, AQP3, AQP4, and AQP7–9) [41,55,117,118,119]. They play a role in transcellular water trafficking from the lumen to the interstitial [118]. AQP8 allows water flow, while AQP3, 7, and 9 also facilitate glycerol flow [119]. It should also be noted that AQP3 is considered among the most important functional molecules in water transport in the colon, whose level of expression in the colon plays an essential role in the laxative effects of osmotic laxatives and stimulant laxatives.

Studies of the AQP expression profile in biopsies of the rectosigmoid mucosa in patients with IBS-D showed that the AQP8 level in ascending and descending colons was significantly lower than in healthy subjects. A differential expression may impair the colonic absorption function in D-IBS patients, reducing colonic fluid absorption and, consequently, forming loose stools and diarrhea. Hence, the change in the distribution of AQP8 could be responsible for diarrhea in patients with IBS-D [41].

Studies on the animal model of IBS found that the expression of AQP1, AQP3, and AQP8 and inflammatory cytokines (IL-1β, TGF-β, and TNF α) was downregulated in the colon of IBS rats. Instead, administering forskolin (5 mg/kg/day for 7 days) via intraperitoneal injection in IBS rats caused an upregulation of AQP1, AQP3, AQP8, and inflammatory cytokines by AMP activation.

Therefore, in light of these data, it can be hypothesized that drugs/molecules with activation action of the cAMP/PKA pathway may play an important role in IBS through the regulation of the release of inflammatory cytokines, the activation of the immune system, and the regulation of the liquid water metabolic abnormalities in the colon [120].

### 3.4. IBS and Ion Channel-Targeting Drugs

Currently, the pharmacological treatment for IBS is targeted toward gastrointestinal guanylyl cyclase C receptors with linaclotide and plecanatide, chloride channels with lubiprostone, transporters with the ion exchanger 3 inhibitor tenapanor, peripheral opioid receptors with loperamide and eluxadoline, gut serotonin receptors with tegaserod and ramosetron, and the gut microbiome using the antibiotic rifaximin [121]. Among these, one of the oldest drugs is lubiprostone, which has been approved by the US Food and Drug Administration (FDA) for treating chronic idiopathic constipation. It is an oral bicyclic fatty acid that selectively activates type 2 chloride channels in the apical membrane of the intestinal epithelial cells, thus stimulating chloride secretion, along with the passive secretion of sodium and water, inducing peristalsis and laxation, without stimulating the GI smooth muscle. Several trials have shown its efficacy in treating chronic idiopathic constipation and IBS-C. Purinergic drugs have also shown promising safety/efficacy profiles for prospective clinical trials in IBD, IBS, functional dyspepsia, and inflammatory diarrhea. Genetic polymorphisms and caffeine consumption may affect susceptibility to treatment [122].

More recently, the available cryoelectron microscopy structure of the palonosetron-bound 5-HT3 receptor that is a ligand-gated K^+^ channel gave the possibility to investigate the binding of palonosetron, granisetron, dolasetron, ondansetron, and cilansetron using molecular dynamics, covering the whole set of antagonists used in clinical practice [123].

Flupirtine, a nonselective KV7 activator used as an antiepileptic drug, inhibited basal Cl^−^ secretion in mouse distal colon and abolished or attenuated the effects of drugs that target various components of enteric neurotransmission. Flupritine did not block the response to epithelium-targeted agents VIP (endogenous VPAC receptor ligand) or carbachol (nonselective cholinergic agonist). Flupirtine inhibited Cl^−^ secretion in both full-thickness and seromuscular-stripped distal colon (containing the submucosal, but not the myenteric plexus) but generated no response in epithelial T84 cell monolayers. KV_7_ channel activators inhibit neuron-driven Cl^−^ secretion in the colonic epithelium and may have therapeutic benefits in treating pathologies associated with the hyperexcitable enteric nervous system, such as irritable bowel syndrome with diarrhea [124].

As reported above, IBS is a common comorbidity in Brugada syndrome type 1 patients. Current migraine and history of migraine are predictors of underlying among patients with FBDs. Frequent coexistence of IBS and Brugada syndrome necessitates cautious use of certain drugs among the therapeutic options for IBS that are known to exacerbate the Brugada phenotype with arrhythmias [125].

Natural mixtures may also act through ion channels. For instance, the Banhasasim-tang, a traditional herbal medicine mixture mainly used to treat GI-related diseases, may have potential in IBS treatment [126]. This mixture inhibited TRPA1, NaV1.5, and NaV1.7 ion channels associated with IBS-mediated visceral hypersensitivity. SiNiSan, a well-known ancient Chinese herbal in the current clinical treatment of irritable bowel syndrome, acts through multiple targets: ADRA2A, HTR2A, F2RL1, F2RL3, PKC, PKA, IL-1Β, NGF, and TRPV1 [127].

## 4. Concluding Remarks

Normal GI function is mainly supported by normal ion channel function; consequently, ion channel abnormalities are linked to GI disease [32]. Patients with an FGID/IBS profile certainly have a complex interaction of susceptible genetic factors, epigenetic factors, and environmental factors.

Several studies have highlighted the role of and strong link between ion channels and mutations affecting ion channels (channelopathies) in FGID/IBS. However, several investigations on many fronts are still needed to understand better the pathophysiology underlying the characteristic FGID/IBS disorders concerning ion channels.

Firstly, a deep investigation of ion channels’ molecular and structural data is needed, and it is crucial to correlate the obtained results with functional data. Secondly, developing new experimental protocols (smooth muscle contraction and its modulation through mechanosensitivity) is mandatory to follow and understand the molecular mechanisms of channelopathies. Lastly, it is necessary to investigate the role of ion channels and their interaction with protein complexes at the GI level.

To better investigate this aspect, studies that integrate data obtained from in vitro research using primary cell systems from the animal models or cell model systems, a family genealogical history of the patient, high-throughput sequencing data, and comparative analyses of the FGID/IBS cohorts with large groups of individuals negative for FGID/IBS should be undertaken. Most GI tissues contain different types of cells, including both excitatory and non-excitable tissues. Determining which cell types or tissues these gene alterations occur would be challenging. In this framework, single-cell analysis would help to solve this issue.

In the context of channelopathies, importance must also be given to the difference in gender; for example, males and females have different probabilities of developing functional anomalies with mutations affecting the same channel. Unfortunately, current databases do not account for this aspect of FGID, making large-scale gender-sensitive comparisons between the genomes of healthy patients and FGID/IBS very difficult.

A better understanding of the role of channelopathies in FGID/IBS patients will offer advances and future avenues for developing effective treatments targeting ion channels addressed to potential personalized therapies.

A limitation of this work is that many statements in the paper actually pertain to inflammatory bowel diseases. Therefore, many of the gene alterations proposed in this paper can be exaggerated. Additionally, many gene alterations do not have functional protein expression.

## Data Availability

Not applicable.

## Abbreviations

AQPs	Aquaporins
FC	Functional constipation
FD	Functional dyspepsia
FGIDs	Functional gastrointestinal disorders
GI	Gastrointestinal tract
IBS	Irritable bowel syndrome
IP	Intestinal permeability
KATP	ATP-sensitive K^+^ channels
TJ	Tight junctions
